# Comprehensive analysis of lysine crotonylation modification in patients with chronic renal failure

**DOI:** 10.1186/s12882-021-02445-4

**Published:** 2021-09-13

**Authors:** Jiahuang Huang, Donge Tang, Fengping Zheng, Huixuan Xu, Yong Dai

**Affiliations:** Clinical Medical Research Center, Guangdong Provincial Engineering Research Center of Autoimmune Disease Precision Medicine, Shenzhen Engineering Research Center of Autoimmune Disease,, The First Affiliated Hospital of Southern University of Science and Technology, Shenzhen People’s Hospital,, Guangdong 518020 Shenzhen, P.R. China

**Keywords:** Crotonylation Modification, Epigenetics, Chronic renal failure, Mass Spectrometry Failure

## Abstract

**Background:**

Post-translational modifications (PTMs) are at the heart of many cellular signaling events, which changes the function of protein. Crotonylation, one of the most important and common PTMs, plays a crucial role in the regulation of various biological processes. However, no study has evaluated the role of lysine crotonylation modification in chronic renal failure (CRF) patients.

**Methods:**

Here, we comparatively evaluated the crotonylation proteome of normal controls and chronic renal failure patients using liquid chromatography-tandem mass spectrometry (LC-MS/MS) coupled with highly sensitive immune-affinity purification.

**Results:**

A total of 1109 lysine modification sites were identified, of which 772 sites were up-regulated and 69 sites were down-regulated. This suggested that crotonylation modification maintains high levels in the patients with chronic renal failure. Gene ontology(GO) enrichment analysis showed that the crotonylated proteins were significantly enriched in the platelet alpha granule lumen, platelet degradulation, and cell adhesion molecule binding. In addition, Kyoto Encyclopedia of Genes and Genomes (KEGG)-based functional enrichment analysis in the Kyoto encyclopedia showed that crotonylated protein was enriched in CD36, which is closely linked to renal failure.

**Conclusions:**

This is the first report of the global crotonylation proteome in chronic renal failure patients. Crotonylation of histone and non-histone may play important roles in delaying the continuous deterioration of renal function in patients with chronic renal failure.

**Supplementary Information:**

The online version contains supplementary material available at 10.1186/s12882-021-02445-4.

## Background

Chronic renal failure (CRF) refers to the abnormal renal function and structure persisting for more than 3 months with or without a decrease in the glomerular filtration rate, and its clinical manifestations vary from asymptomatic, laboratory abnormalities to uraemia [1]. The global prevalence rate of CRF has reached 14.3 %. Recently, the incidence of CRF, especially terminal-stage renal diseases, has significantly increased, which is a serious threat to human health [1, 2]. Indeed, Chronic renal failure was among the fastest-growing causes of death worldwide and estimated to become the second most common cause of death within the next century in some countries [2]. Renal interstitial fibrosis is a histological feature of CRF and an important predictor of renal function loss in patients [3]. It has been demonstrated that Post-translational modifications (PTMs) can form epigenetic layers that respond to environmental signals and external stimuli, thereby altering the expression of genes involved in CRF [4]. Epigenetic changes, including the importance of PTMs in fibrosis, inflammation and immunity related to various kidney diseases, are becoming more important in the development of CFR [5].

PTMs are an essential part of the protein maturation process, which changes the functions of proteins [6, 7]. Improvements in the mass spectrometry (MS) technique enhance proteome research and contribute to the recognition of a rich list of PTMs [8]. Protein lysine acetylation is a ubiquitous and reversible PTM. The initial results were mainly through the catalytic regulation of gene transcription and expression in the nucleus by histone acetyltransferase and histone deacetylase [9]. In chromatin biology, lysine acetylation was discovered in tubulin and mitochondrial proteins, suggesting that lysine acetylation significantly contributes to cell biology [10]. Besides lysine acetylation, some new types of PTMs, such as lysine malonylation and succinylation, have been identified and play an important role in regulating various eukaryotic and prokaryotic physiological functions [11, 12].

Lysine crotonylation, a novel protein PTM, was initially found in human cell lines and mouse sperm histone [13]. The discovery of lysine crotonylation has attracted extensive attention to the scientific circle. Lysine crotonylation has been deeply studied in a short time. Lysine crotonylation has been demonstrated first as a potent indicator of active promoters and could be a major signal for controlling male germ cell differentiation [14]. Wei et al [15] has believed that DNA replication may be affected by protein crotonylation, which may result in the inhibition of DNA replication, thus affecting the cell cycle. All studies have suggested that lysine crotonylation controls the interpretation of genetic data at the chromatin level and plays a major role in gene expression and cell fate.

Histone acetylation has been adequately characterised in this paradigm. So, far, studies on histone acetylation have focused on tumours, neuropsychiatric disorders, lupus, cardiovascular diseases, acute lymphoblastic leukaemia, diabetic nephropathy, acute kidney injury (AKI) and chronic nephropathy [1, 16–21]. In addition, histone acetylation shares the same enzyme system as histone crotonylation [22]. According to this, the histone acetylation modification group has a structure similar to that of the lysine crotonylation group [14]. Recent studies have shown that histone deacetylase inhibitors have protective effects on some experimental models of renal injury [23, 24]. Histone crotonylation has been observed in renal tissue, suggesting that it contributes to the epigenetic regulation of gene expression during renal injury [25]. This study found that lysine crotonylation in renal tissue increased during AKI, and crotonate supplementation protected healthy renal tissue from nephrotoxic AKI [26]. Therefore, we speculate that lysine crotonylation significantly contributes to various biological processes and is closely related to the pathogenesis of CRF. However, data on the relationship between lysine crotonylation and CRF are limited, and the pathogenesis of CRF remains unclear. Most studies have focused on histone crotonylation and its function, but recent studies have shown that lysine crotonylation also occurs in several non-histone proteins [27]. This suggests that non-histone proteins are also widely present in the human body and play a related function, but data on this aspect are still lacking. Therefore, we speculate that lysine crotonylation is closely associated with the pathogenesis of renal failure and related changes in non-histone proteins.

In this study, highly sensitive immune affinity purification and high-resolution liquid chromatography tandem mass spectrometry (LC-MS/MS) were used to examine quantitative proteomics. A quantitative assessment on crotonylated proteomics was conducted in this study. Of 347 proteins, 1,109 lysine crotonylation sites were identified. The identified crotonylated proteins were mainly located in the cytoplasm, nucleus, mitochondria and extracellular region. The identified crotonylated proteins were primarily localised in the cytoplasm, nucleus, mitochondria and extracellular region. Bioinformatic analysis was performed to reveal the biological functions of crotonylated proteins. Further bioinformatics analysis showed that the biological function of crotonylated proteins was significantly enriched on CD36 of platelets and blood cells. As far as we know, this is the first study describing lysine crotonylation in the global proteome of CRF, thus expanding the understanding of the role of lysine crotonylation in the pathophysiological process of patients with CRF.

## Materials and methods

### Sample collection

The peripheral blood samples of 6 patients with chronic renal failure and 6 healthy blood donors were collected and tested. Those six patients were diagnosed with chronic renal failure (CKD 5-stage GFR<15ml/min) by Shenzhen People’s Hospital and the primary etiology of CRF was glomerulonephritis. The healthy controls freed from any diseases, including cancer, allergies, diabetes, or infectious diseases. All patients and healthy blood donors were over 18 years old (Table 1), and signed consent before this study. And it was approved by the Ethics Committee of Clinical Research of Shenzhen People’s Hospital. After sample collection, peripheral blood mononuclear cells (PBMCs) were isolated by density gradient centrifugation using Ficoll-Hypaque (GE Healthcare Bio-sciences AB, Uppsala, Sweden) according to the manufacturer’s protocol.PBMCs were lysed with TRIzol reagent (Invitrogen, Carlsbad, CA) and stored at − 80 °C.

**Table 1:** Clinical characteristics of patients and normal controls

Group	number	sex		age
		man	woman	
CRF	6	4	2	39.78 ± 11.07
NC	6	4	2	42.22 ± 12.41

### Protein extraction

Sample was sonicated thrice on ice using a high intensity ultrasonic processor (Scientz) in a lysis buffer (8 M urea, 1 % protease inhibitor cocktail). After centrifugation at 12,000 ×g and 4 °C for 10 min. The supernatant was collected and protein concentration was determined with the bicinchoninic acid kit (Sigma Chemical Co., St. Louis, MO) according to the manufacturer’s instructions.

### Trypsin digestion

For digestion, the protein solution was reduced with 5 mM dithiothreitol for 30 min at 56 °C and alkylated with 11 MM iodoacetamide for 15 min at room temperature in darkness. The protein sample was then diluted by adding 100 mM TEAB to urea concentration less than 2 M. Finally, trypsin was added at 1:50 trypsin-to-protein mass ratio for the first digestion overnight and 1:100 trypsin-to-protein mass ratio for a second 4 h-digestion.

### Tandem mass tag/isobaric tags for relative and absolute quantitation (TMT/iTRAQ) labeling

After trypsin digestion, peptide was desalted by Strata X C18 SPE column (Phenomenex) and vacuum-dried. Peptide was reconstituted in 0.5 M TEAB and processed according to the manufacturer’s protocol for TMT kit/iTRAQ kit. Briefly, one unit of TMT/iTRAQ reagent were thawed and reconstituted in acetonitrile. The peptide mixtures were then incubated for 2 h at room temperature and pooled, desalted and dried by vacuum centrifugation.

### High-performance liquid chromatography (HPLC) fractionation

The tryptic peptides were fractionated into fractions with high pH reverse-phase HPLC using Thermo Betasil C18 column (5 μm particles, 10 mm ID, 250 mm length). Briefly, peptides were first separated with a gradient of 8–32 % acetonitrile (pH 9.0) over 60 min into 60 fractions. The peptides were then combined into 6 fractions and dried by vacuum centrifugation.

### Affinity enrichment

To enrich Kcr-modified peptides, tryptic peptides dissolved in NETN buffer (100 mM sodium chloride [NaCl], 1 mM ethylenediaminetetraacetic acid [EDTA], 50 mM Tris–HCl, and 0.5 % NP-40, pH 8.0) were incubated with pre-washed antibody beads (PTM Bio) at 4 °C overnight with gentle shaking. The beads were washed 4 times with NETN buffer and twice with water. The bound peptides were eluted from the beads with 0.1 % trifluoroacetic acid. The eluted fractions were combined and vacuum dried. For LC-MS/MS analysis, the resulting peptides were desalted with C18 ZipTips (Millipore) according to the manufacturer’s instructions.

### LC-MS/MS analysis

The tryptic peptides were dissolved in 0.1% formic acid (solvent A), directly loaded onto a home-made reversed phase analytical column (15 cm length , 75 μm i.d.). The gradient was comprised of an increase from 6% to 23% solvent B (0.1% formic acid in 98% acetonitrile)over 26 min, 23% to 35% in 8 min and climbing to 80% in 3 min then holding at 80% for the last 3 min, all at a constant flow rate of 400 n L /min on an EASY nLC 1000 UPLC system.

The peptides were subjected to NSI source followed by tandem mass spectrometry (MS/MS) in Q Exactive TM Plus (Thermo) coupled online to the UPLC. The electrospray voltage applied was 2.0 kV. The m/z scan range was 350 to 1800 for full scan, and intact peptides were detected in the Orbitrap at a resolution of 70,000. Peptides were then selected for MS/MS using NCE setting as 28 and the fragments were detected in the Orbitrap at a resolution of 17,500. A data dependent procedure that alternated between one MS scan followed by 20 MS/MS scans with 15.0s dynamic exclusion. Automatic gain control (AGC) was set at 5E4. Fixed first mass was set as 100 m/z.

### Database search

The resulting MS/MS data were processed using MaxQuant search engine (v.1.5.2.8). Tandem mass spectra were searched against the human database concatenated with a reverse decoy database. Trypsin/P was specified as the cleavage enzyme, allowing up to 4 missing cleavages. The mass tolerance for precursor ions was set as 20 ppm in the first search and 5 ppm in the main search, while that for the fragment ions was set as 0.02 Da. Carbamidomethylation on Cys was specified as fixed modification and oxidation on Met was specified as variable modification. False discovery rate (FDR) was adjusted to < 1 % and the minimum score for modified peptides was set as > 40.

### Gene ontology (GO) annotation

Gene ontology annotation was derived from the UniProt-GOA (http://www.ebi.ac.uk/GOA/). The identified protein ID was converted to UniProt ID, followed by its mapping to GO IDs using the protein ID. If some identified proteins were not annotated By UniProt-GOA, InterProScan software (http://www.ebi.ac.uk/interpro/search/sequence-search) was used to annotate protein’s GO functions, based on protein sequence alignment method. The proteins were GO annotated based on the following 3 categories: biological process, cellular component, and molecular function. For each category, a two-tailed Fisher exact test was employed to test the enrichment of the differentially expressed protein against all identified proteins. GO with a corrected value of *P* <0 0.05 was considered significant.

### Protein domain annotation

The domain functional description of the identified proteins was annotated by InterProScan (a sequence analysis application) based on the protein sequence alignment method using InterPro (http://www.ebi.ac.uk/interpro/) domain database. For each category of proteins, InterPro database was searched and a two-tailed Fisher exact test was used to test the enrichment of the differentially expressed protein against all identified proteins. Protein domains with a corrected value of *P* <0 0.05 were considered significant.

### KEGG channel annotation

We used KEGG online service tool KAAS (http://www.genome.jp/tools/kaas/) to annotate protein’s KEGG database description. The mapping of the annotation result on the KEGG pathway database was performed using KEGG online service tool KEGG mapper. The pathway with a corrected value of *P* < 0 0.05 was considered significant. These pathways were classified into hierarchical categories according to KEGG website.

### Subcellular localization

We used WoLF PSORT (http://www.genscript.com/wolf-psort.html), a subcellular localization predication software to predict subcellular localization.

### Motif analysis

Soft motif x was used to analysis the model of sequences constituted with amino acids in specific positions of modify 21 mers (10 amino acids upstream and downstream of the site) in all protein sequences. And all the database protein sequences were used as background database parameter, other parameters with default.[hj1] [hj1]To improve description of experimental procedures.

## Result

### Comparative analysis on complete protein and lysine crotonylation in patients with chronic renal failure and healthy controls

**Fig. 1:**
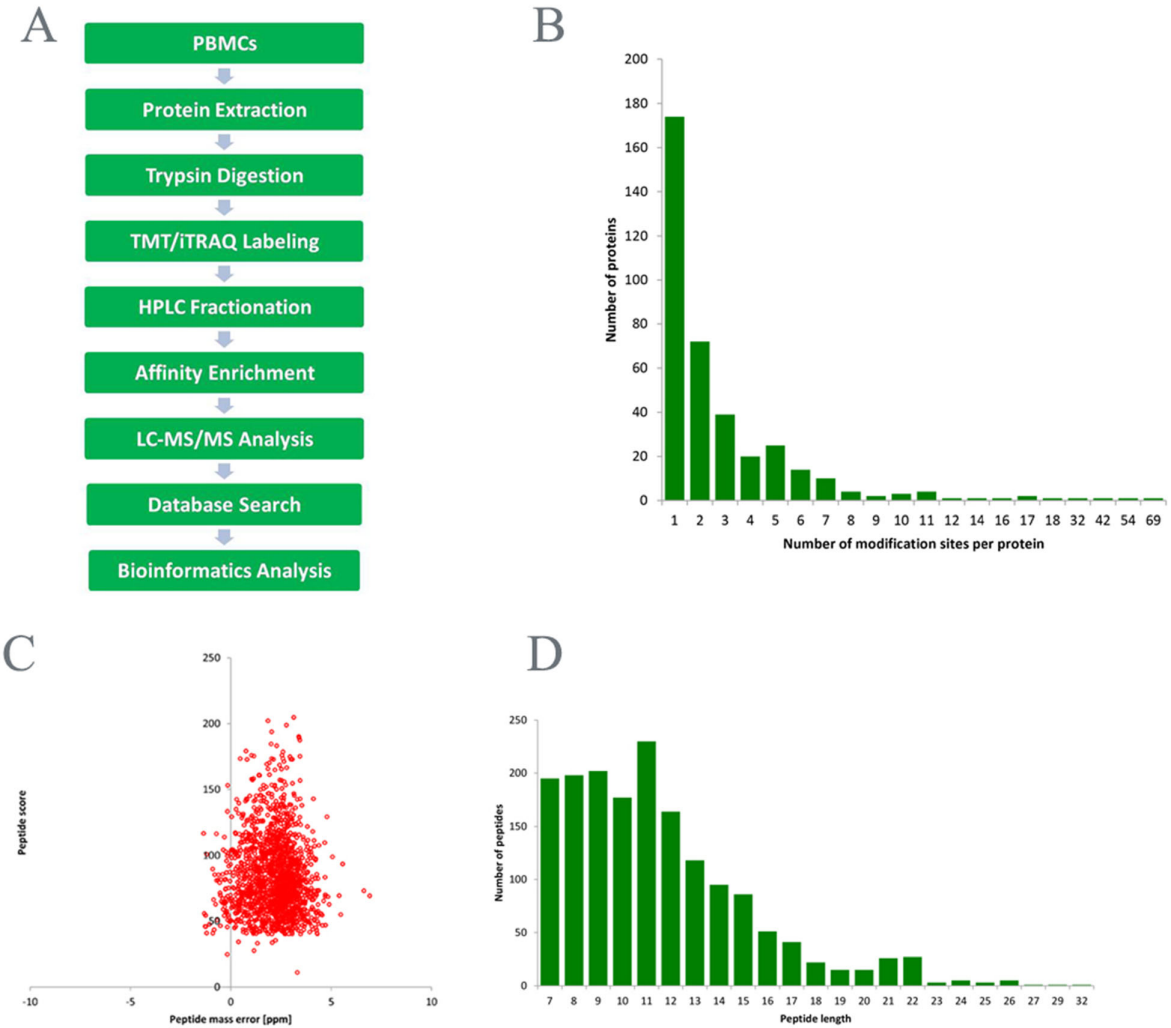
Comparatively analysis of whole proteome and lysine crotonylation between CRF and NC. (**A**) Overview of experimental procedures used in the presentstudy. (**B**) Mass error distribution of all crotonylated peptides. (**C**) Distribution of lysine crotonylation in one protein. (**D**) Distribution of lysine crotonylation peptides based on their length

The whole process consists of nine steps (Fig. 1 A). A total of 1209 crotonylation sites were identified, of which 1109 of 347 proteins were quantifiable (Table S1). As fold changes over 1.2 as an up regulation and below 1/1.2 as a down regulation. There was a differential expression of 260 proteins in chronic renal failure and healthy controls, of which 772 sites were from up-regulated 260 proteins and 51 sites from 69 down-regulated proteins. Another result showed that significantly differential expression lysine crotonylation between the CRF and NC (Table S2). In order to verify the validity of mass spectrum data, the mass errors of all identified peptides were evaluated. The quality error is centered at 0 and below 10 ppm, which shows that the quality accuracy of MS data meets the requirement (Fig. 1B). Among 347 crotonylated proteins, most proteins contain one or two crotonylation sites, while fewer proteins have 7 or more crotonylation sites (Fig. 1 C). The length of most peptides varied from 8 to 20 amino acids, consistent with the rule of trypsin digestion ( Fig. 1D).

### Analysis of crotonylation site motif

**Fig. 2:**
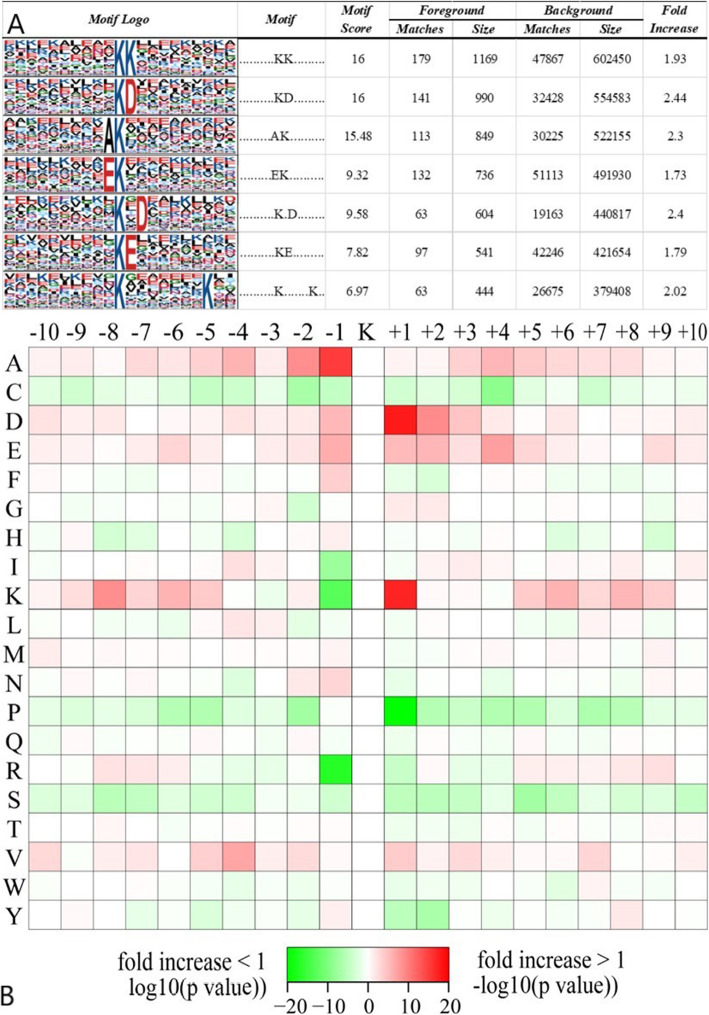
Analysisof crotonylation site motif. (**A**) Sequence probability logos of significantly enriched crotonylation site motifs around the lysine crotonylation sites. (**B**) Heat map of the amino acid compositions around the down-regulated lysine crotonylation sites showing the frequency of different types of amino acids around this residue. Red indicates enrichment and green indicates depletion

In order to understand the sequence commonness around crotonylation site and compare it with acetylated site, the sequence motifs of all identified crotonylated peptides were studied by Motif-X program. In total, 7 conserved motifs (KK, KD, AK, EK, K.D, Ke, K.K) were retrieved (Table S3, Fig. 2 A). Particularly, the motifs of Ke and KD are very conservative. It is important that the amino acids that are significantly conserved among these motifs, E and D, are negatively charged and are rarely found in other PTMS. These motifs may represent a characteristic of crotonylation in chronic renal failure. Hierarchical clustering analysis is also worked out to further analyze these topics (Fig. 2B). At − 10 to − 5 and 10 to 5, the positively charged K residue is enriched, while the negatively charged residues D and E are significantly enriched at the − 1 to 4 position. Residues of short aliphatic groups often appeared at the − 7 ~ 8 position, but sulfur-containing C residues were not found.

### Functional classification of crotonylation in GO and crotonylation subcellular localization

**Fig. 3:**
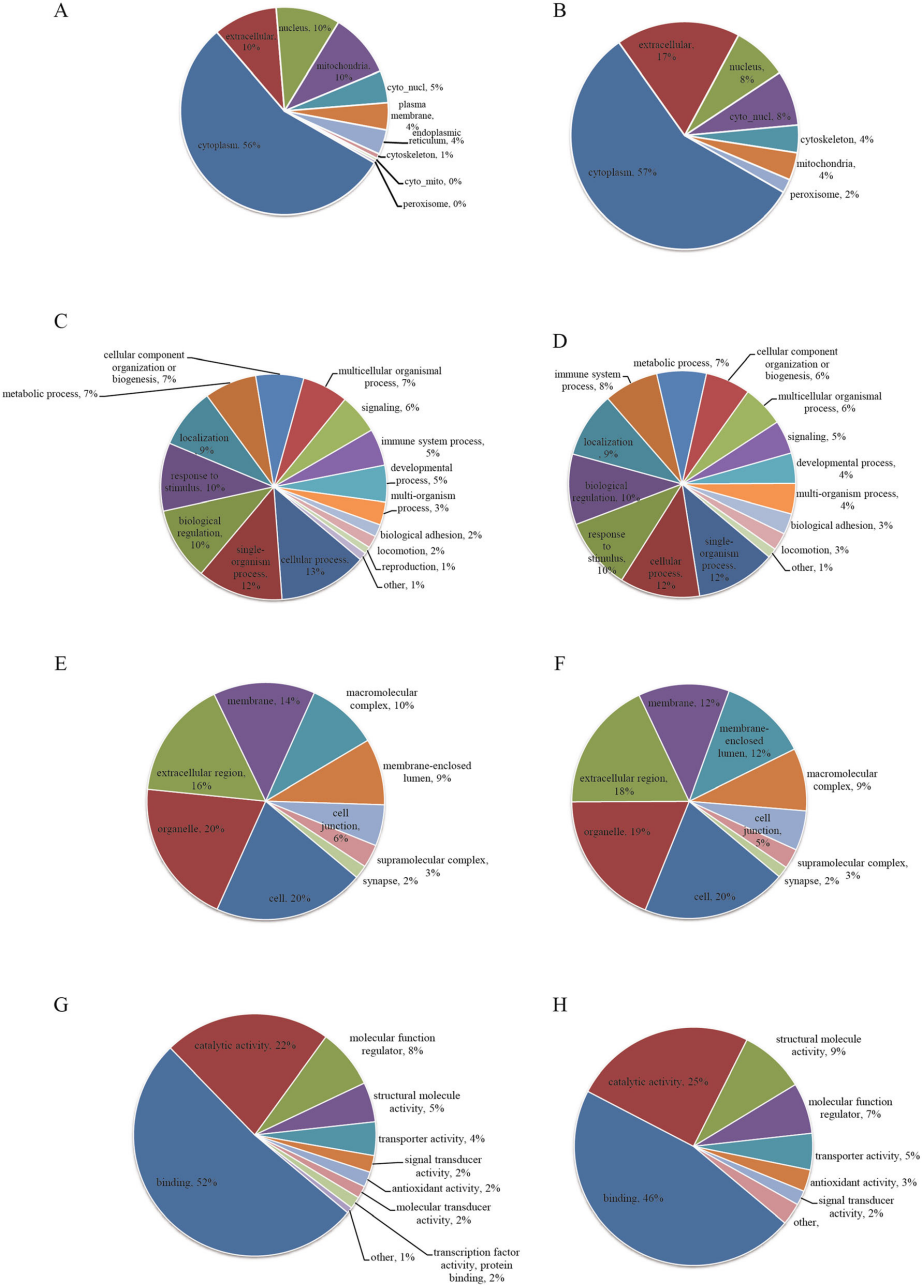
Functional classification of lysine crotonylation in GO and subcellular. (**A**) and (**B**) Subcellular localization of upregulated and downregulatedcrotonylatedproteins. (**C**) and (**D**) GO- analysis for upregulated and downregulated crotonylated proteins in biological processes. (**E**) and (**F**) GO- analysis for upregulated and downregulated crotonylated proteins in cellular component. (**G**) and (**H**) GO- analysis for upregulated and downregulated crotonylated proteins in molecular function

Functional classification of GO and subcellular lysine crotonylation the subcellular localization characteristics of lysine crotonylation have been identified (Fig. 3 A). It turned out that up-regulated proteins were mainly distributed in cytoplasm (56 %), nucleus (10 %), mitochondria (10 %) and extracellular (10 %), while down-regulated proteins were mainly distributed in cytoplasm (57 %), nucleus (7 %), mitochondria (4 %) and extracellular (17 %) (Fig. 3B). It showed that there was no significant difference in protein localization between up-regulated and down-regulated proteins. In order to understand the general situation of crotonyl protein in chronic renal failure, on the basis of its biological process, molecular function and cell composition, the GO functional classification of all crotonylates proteins was studied (Table S4). In the category of biological process, most crotonylated proteins are associated with cellular processes, single organism processes, biological regulation and stimulation (Fig. 3C), and most down-regulated proteins are associated with cellular processes, single organism processes, biological regulation and stimulation (Fig. 3D). In cell components, most crotonylated proteins are associated with cells, organelles, extracellular domains, and the membrane of up-regulated proteins (Fig. 3E), while most of the down-regulated proteins are associated with cells, organelles, extracellular domains and membranes (Fig. 3 F). In the molecular functional category, most crotonylated proteins are associated with up-regulated protein binding, catalytic activity, structural molecular activity and molecular function modulators (Fig. 3G), while most down-regulated proteins are concerned with binding, catalytic activity, molecular function modulators and structural molecular activity (Fig. 3H). There was no significant difference in GO functional classification between up-regulated and down-regulated proteins, suggesting that crotonylation of lysine might have a large scale of biological functions.

### Functional enrichment of Kcr in GO, KEGG, and protein domain

**Fig. 4:**
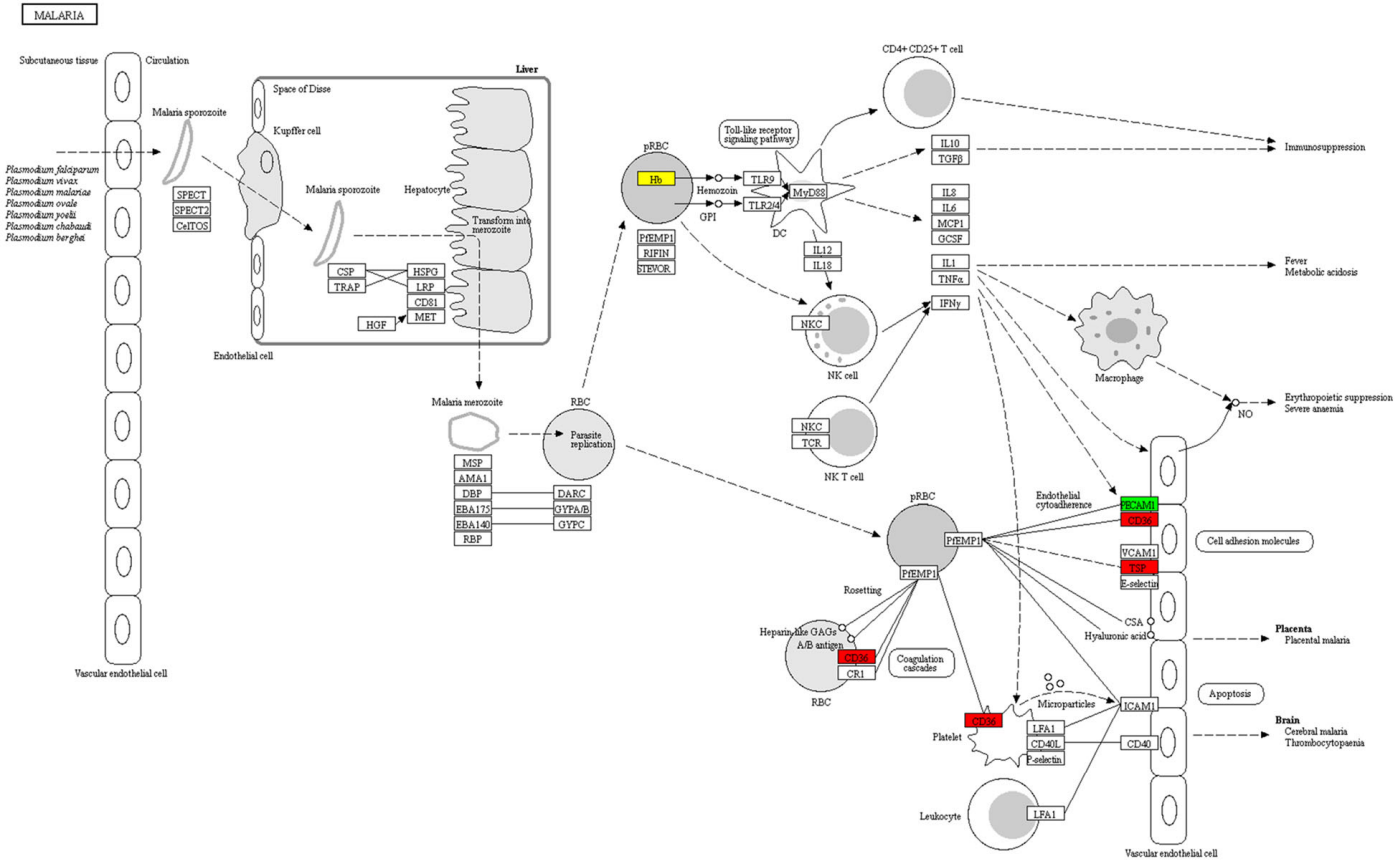
(**A**) Protein-protein interaction Network of carbon Metabolism Pathway. (**B**) Visual display of significant enrichment of proteins corresponding to differential modification sites in a KEGG pathway (CRF/NC). The modification level upregulation protein is indicated by red; the modification level down-regulation protein is expressed by bright green; and there are several proteins in the node expressed by yellow, which contain the up-regulated and down-regulated proteins at the modification level. (**C**) quantified crotonylated proteins were divided into four quantiles

**Fig. 5:**
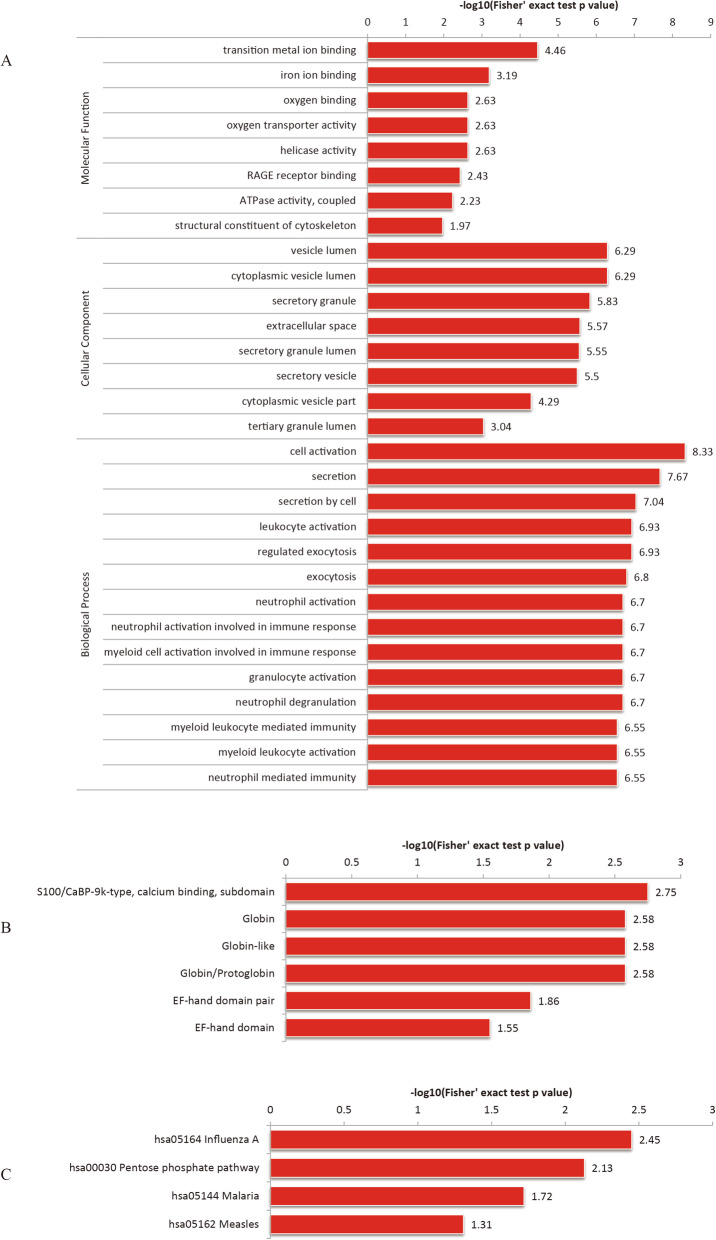
(**A**) Protein-protein interaction Network of carbon Metabolism Pathway. (**B**) Visual display of significant enrichment of proteins corresponding to differential modification sites in a KEGG pathway (CRF/NC). The modification level upregulation protein is indicated by red; the modification level down-regulation protein is expressed by bright green; and there are several proteins in the node expressed by yellow, which contain the up-regulated and down-regulated proteins at the modification level. (**B**) quantified crotonylated proteins were divided into four quantiles

**Fig. 6:**
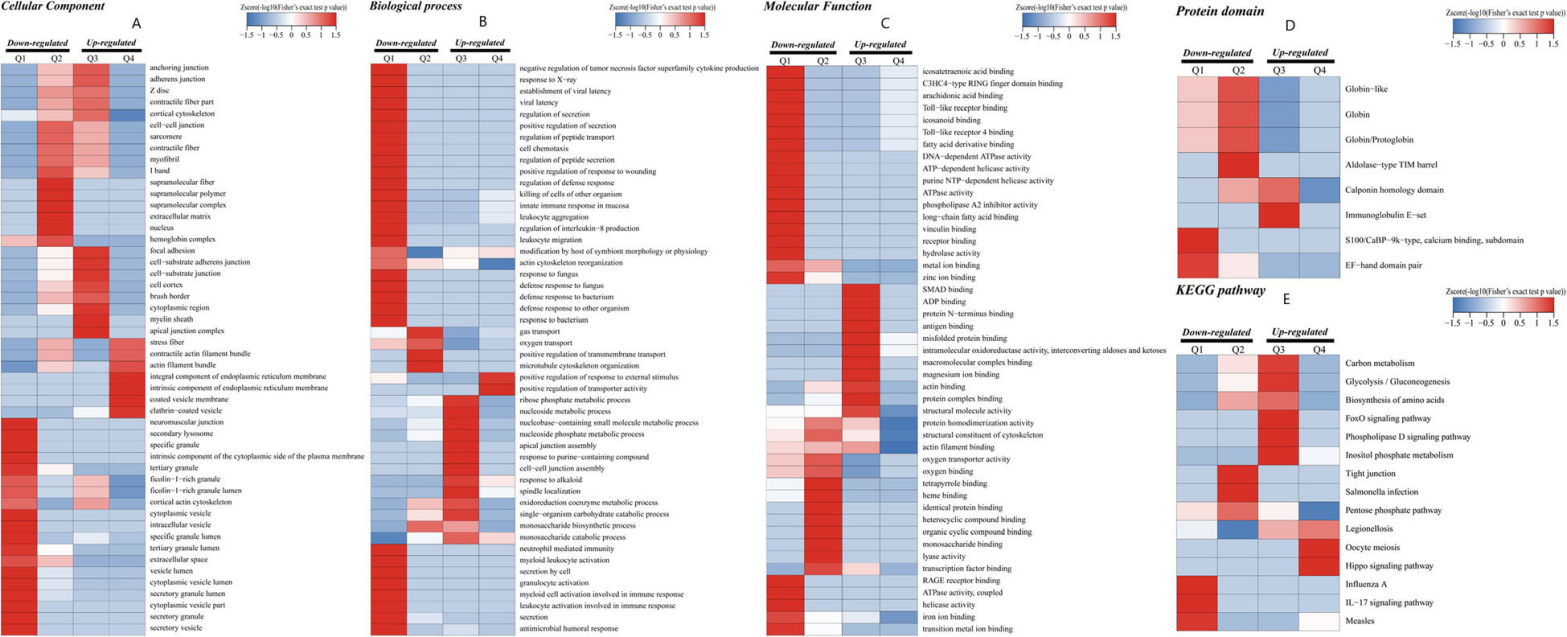
???

The functional enrichment of GO, KEGG and protein domain lysine crotonylation based on GO was studied (Table S5). Highly expressed crotonylated protein is highly enriched on platelets and erythrocytes CD36 (Fig. 4). The down-regulation of crotonylated protein mainly includes those (Fig. 5A). At the same time, the function enrichment analysis based on KEGG was carried out (Table S6). It was found, however, that there was no up-regulated crotonylated protein in KEGG (Fig. 5B). In addition, the down-regulated crotonylation domain includes the S100/CaBP9K calcium binding subdomain, globin, globin-like, Globin/Protoglobin. The EF-hand domain pair and EF-hand domain (Table S7, Fig. 5C) suggest an important part for crotonylation during these processes. Similarly, there was no up-regulated crotonylated the protein domain in this study. Pentose phosphate pathway has an important relationship with complications of CRF. Search for crotonylated involved in carbon metabolism, including dense protein interaction networks (Fig. 6A).

### Cluster analyses in GO, KEGG, and protein domain

For the sake of understanding the function of lysine crotonylation in more detail, we performed GO, KEGG, and protein domain enrichment-based clustering analyses. All quantized crotonylation sites were separated into four quartiles according to the multiple changes of the Lysine crotonylation sites: Q1 (0<−<0.77), Q2 (0.77 < 0.77), Q3 (1.2<−<1.3), and Q4 (ratio > 1.3), P value < 0.05. Q1, Q2, Q3 and Q4 have 54, 15, 98 and 647 crotonylation, respectively. The four kinds of quantifiable proteins were analyzed by cluster analysis. Q1 and Q2 are considered down-regulated, while Q3 and Q4 are considered up-regulated (Fig. 6B).

**Fig. 7:**
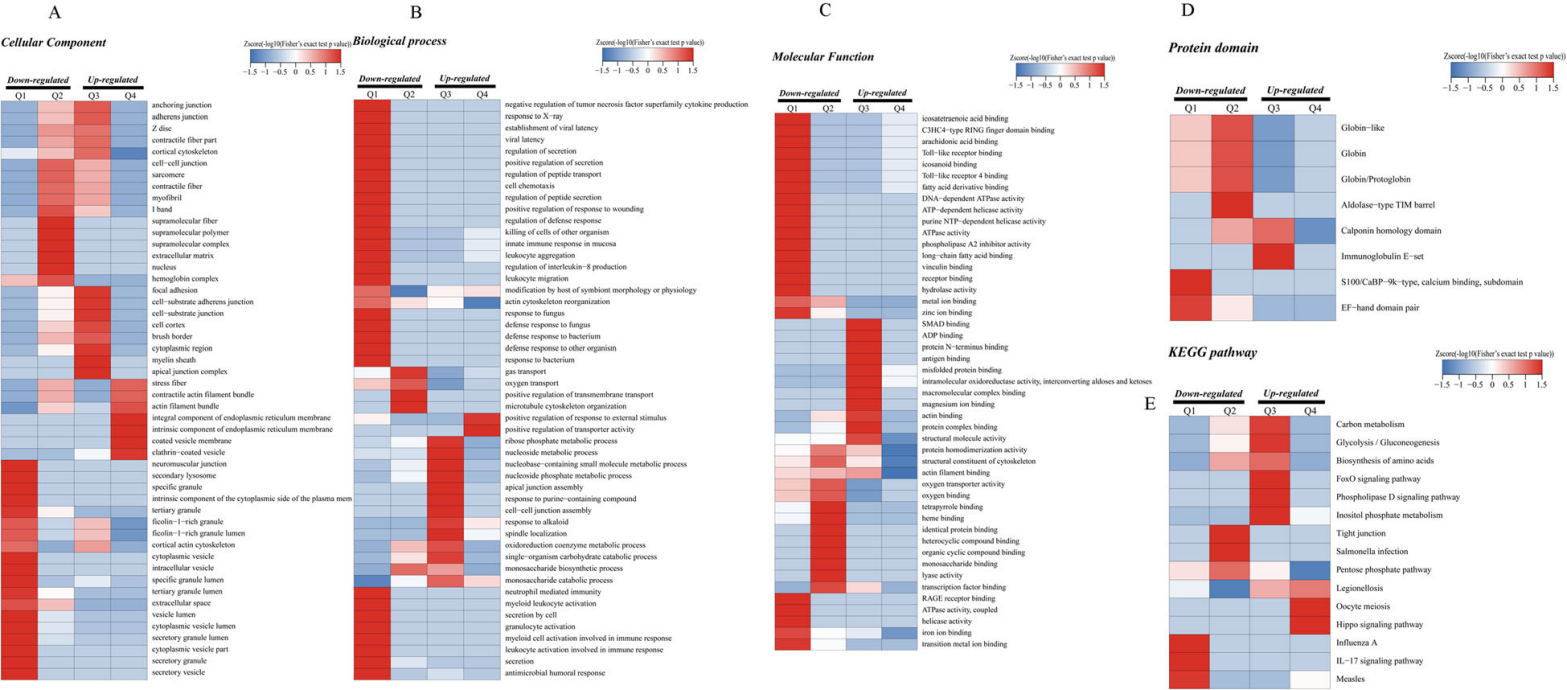
Functional enrichment of lysine crotonylation in GO, KEGG,and proteindomain. (**A**) GO-based enrichment analysis of upregulated crotonylated proteins in terms of cellular component, molecular function, and biological process. (**B**) KEGG-based enrichment analysis of upregulated crotonylated proteins. (**C**) Protein domain- based enrichment analysis of upregulated crotonylated proteins

For GO analysis, it was found that the crotonylated protein in Q1 was mainly enriched in cell secretion, and the crotonylated protein in Q2 was mainly enriched in cell substrates, neurons and cytoskeleton, the crotonylated protein in Q3 was mainly enriched in cell junctions, while the crotonylated protein in Q4 was mainly abundant in cell microcrystals composed of cell membranes and cells (Fig. 7A). In addition, the biological enrichment process of crotonylation was also carried out. It was found that the crotonylated protein in Q1 was mainly concentrated in the cell response to pathophysiology, and that the crotoacylated protein in Q2 was located in cell function, such as migration and development. However, the crotonylated in Q3 is mainly enriched in cell metabolism. In addition, the crotonylated protein in Q4 is mainly concentrated in the cell response to the outside world (Fig. 7B). For the molecular functional crotonylated proteins, they were found to be highly enriched in the activity of cells in Q1 and highly increased in the cell-binding processes of Q3 and Q4 (Fig. 7C).

The enrichment of KEGG suggests that certain pathways of Q3 and Q2 are associated with diseases, for instance, pancreatic cancer, type II diabetes, cellular metabolism, and salmonella infection, while crotonylated protein is abundant in cell signaling pathways in Q1 and measles and influenza viruses, and crotonylated protein is enriched in cell signaling pathways and germ cell division in Q4 (Fig. 7D).

At the same time, the protein domain of crotonylated protein was studied. They were found to be highly enriched in Q2: Calponin homology domain, aldolase-Tim type barrel, globin/proton protein, globin, globin-like (Fig. 7E).

### Protein–protein interaction network of the Kcr proteins

**Fig. 8:**
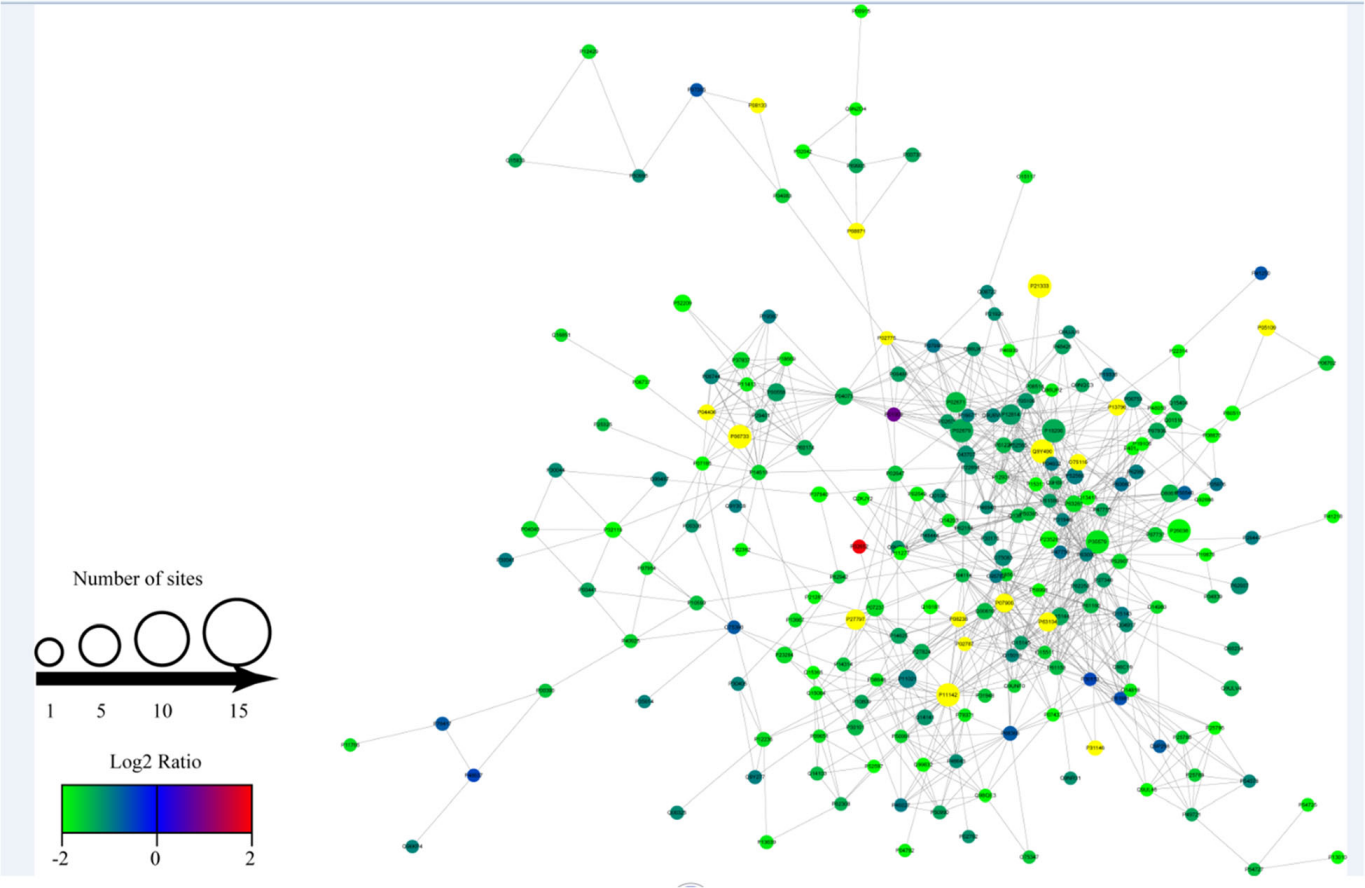
Clustering analysis in GO, KEGG and protein domain. (**A**)Cellular component analysis. (**B**)Biological processanalysis. (**C**) Molecular function analysis. (**D**)KEGG pathway analysis. (**E**) protein domain

To further identify the cellular processes regulated through crotonylation in CRF protein-protein interaction network of the Kcr proteins was established (Fig. 8). A total of 888 pairs of protein-protein mapping to the protein interaction database, showing the different cellular function of crotonylated protein in CRF. The physiological interaction between these protein complexes may contribute to their synergy and coordination in CRF.

## Discussion

In this study, a comparative study was conducted between the healthy control group and the crotonylated proteome of CRF, and the crotonylated peptide was identified using the antibody affinity enrichment method. High-resolution MS was performed. The method identified 1,209 crotonyl sites on 377 proteins, of which 1,109 sites in 347 proteins contained quantitative information. The modification level of 772 loci was upregulated, and the modification level of 69 loci was downregulated in the CRF/healthy control group. The roles of these different proteins in cellular and molecular functions and the related signalling pathways were discussed by gene ontology (GO) and Kyoto Encyclopedia of Genes and Genomes (KEGG) analyses, which laid a foundation for the study of the distribution of apparent lysine crotonylation modification in higher biological eukaryotes.

This study was the first attempt to describe lysine crotonylation in patients with CRF. In the past, there has been evidence of the role of PTM mutations in CRF. Fibrosis, cell cycle and the abnormal expression of inflammatory genes are the key events in the progression of CRF and are related to the changes in PTMs [28]. Non-histone crotonylated proteins exist widely in the human body and play related roles [29]. According to the aforementioned data, the crotonylation levels of histone and non-histone proteins increased in patients with CRF.

A recent study has found that lysine crotonylation in the kidney increases during AKI. Lysine crotonylation may play a role in the repair of AKI. Because lysine crotonylation in renal tubular cells and kidneys in vitro increases the expression of PGC-1α and sirtuin-3 that have neuroprotective actions on cultured renal tubular cells and normal kidneys and downregulates genes involved in tissue damage, such as encoding MCP-1 chemokines of CCL2, a chemokine that contributes to kidney PGC-1α inflammation [30]. Parenteral injection of crotonic acid increases the level of crotonylation in renal tissue and reduces inflammatory markers, mitochondrial stress, renal dysfunction and renal damage through excessive doses of experimental aldosterone protection [31]. These results suggest that lysine crotonylation is beneficial to the recovery of AKI. AKI and CRF share several pathogenic processes, including inflammation and parenchymal cell death [30].They are considered interconnected syndromes, as CRF predisposes affected patients to AKI, and AKI may accelerate CRF progression. Thus, lysine crotonylation also can slow the progression of CRF and AKI. However, we not only found that crotonylation of histone proteins is increased but also found that crotonylation of non-histone proteins is increased and was enriched in CD36 in patients with CRF; this is not found in AKI. Crotonylated non-histone proteins have been characterised in different cell types, such as Hela and H1299 cells, and in tissues, such as the lung, kidney, liver, colon, uterus, ovary and brain of mice [32]. It is thought to be important in various biological processes, such as RNA splicing, gene expression, chromatin organisation, nucleic acid metabolism and cell cycle. However, the requirement of crotonylation for most putative cellular functions has not been formally tested.

The functional enrichment of lysine crotonylation in GO showed that crotonylated proteins are related to many biological processes, including cellular structural components and cellular molecular binding and pathophysiological processes involved in these biological processes, suggesting that the various interactions related to these biological processes may be regulated by modifying proteins. The functional enrichment of lysine crotonylation in KEGG was studied. However, only the B-class scavenger receptor CD36, a KEGG pathway, is closely related to renal failure. It showed that crotonylated proteins were enriched in CD36. CD36 is a multifunctional receptor that mediates the binding and cellular uptake of long-chain fatty acids, oxidised lipids and phospholipids, advanced oxidation protein products, thrombospondin and advanced glycation end products and contributes to lipid accumulation, inflammatory signalling, energy reprogramming, apoptosis and kidney fibrosis [33]. Furthermore, it is expressed in various kidney cells, such as proximal tubular epithelial cells, mesangial cells, podocytes, monocytes and macrophages [32]. To promote CRF, CD36 is involved in lipid accumulation, inflammation, energy reprogramming, apoptosis and kidney fibrosis by activating Toll-like receptors, Na+/K + ATPase, NLRP3 inflammasome, PKC-NAPDH oxidase, Scr/Lyn/Fyn and mitogen-activated protein kinases and TGF-β signalling pathways [32]. Recent studies have shown that in patients with diabetes, hyperglycemia can increase the expression of CD36, aggravate platelet-mediated inflammation [34], cause apoptosis of renal tubular epithelial cells and accelerate renal tubular degeneration and renal interstitial fibrosis [35]. Thus, experimental studies have demonstrated that blockade or knockout of CD36 prevents kidney injury. Selected patients with CRF have shown elevated soluble fibrin plasma levels and enhanced thrombin-induced thrombin generation, which was normalised by CD36 blocking, suggesting that CD36 is a novel therapeutic target for preventing kidney fibrosis [36]. The expression and intracellular location of CD36 are regulated by multiple ligands that contribute to gene transcription and PTMs. Histone crotonylation can promote the expression of genes in the range of increasing substrate availability, but under the condition of promoting inflammation or cell stress, histone crotonylation may decrease the expression of some genes, which are mainly enriched in CD36 [37, 38]. We can speculate that histone crotonylation affects the expression of CD36-related genes.

In another study, data have shown that crotonylation-modified proteins were reduced in haemodialysis patients because crotonylation may contribute to the recovery of AKI. However, patients who maintain haemodialysis are unlikely to restore renal function [39], combined with the role of crotonic acid modification currently being studied in kidney cells. We can speculate that crotonylation also alleviates disease progression in patients with CRF and restores some of its functions, whereas in patients with CRF, histone and non-histone proteins are higher than those in healthy controls. It can be speculated that in some ways, the body modifies histone and non-histone protein to delay renal fibrosis, further restore renal function and delay the progression of CRF.

Presently, the aetiology of CRF is complicated, the clinical manifestations are diversified, the treatment is difficult and the prognosis is of a sort. However, the current treatment is limited. Few drugs can inhibit the progression of CRF. By further studying the lysine crotonylation reaction in the global proteome of CRF, its function in the progression of CRF can be further understood. The current therapeutic drugs for post-translational modification of histone, even if they may affect the expression of multiple genes with different or even opposite functions, can also have certain positive effects under various pathological conditions, including renal injury [40]. If the mechanism of lysine crotonylation modification in CRF was studied, by changing the crotonylation modification to achieve the purpose of preventing and treating diseases, it provides a new idea for treating CRF.

## Supplementary Information

**Additional file 1: Table S1.** Detailed information for all identified crotonylated peptides and their corresponding proteins.

**Additional file 2: Table S2.** Significantly differential expression lysine crotonylation between the CRF and NC.

**Additional file 3: Table S3.** The seven motifs retrieved from lysines crotonylated peptides.

**Additional file 4: Table S4.** GO annotation detail of the crotonylated proteins.

**Additional file 5: Table S5.** GO-based enrichment analysis of crotonylated proteins in terms of cellular component, molecular function, and biological process.

**Additional file 6: Table S6.** KEGG pathway-based enrichment analysis of crotonylated proteins.

**Additional file 7: Table S7.** Protein domain enrichment analysis of crotonylated proteins.

## Data Availability

The data of the current study are available from the corresponding author on reasonable request.
